# Telomeric repeat-containing lncRNA TERRA targets non-telomeric DNA in trans via R-loops

**DOI:** 10.1016/j.csbj.2025.11.063

**Published:** 2025-11-29

**Authors:** Saifeldin N. Shehata, Akram Mendez, Tanmoy Mondal, Roshan Vaid

**Affiliations:** a Department of Laboratory Medicine, Institute of Biomedicine, University of Gothenburg, Gothenburg, Sweden; b Division of Cellular Medicine, Jacqui Wood Cancer Centre, School of Medicine, University of Dundee, Scotland, UK; c Region Västra Götaland, Sahlgrenska University Hospital, Department of Clinical Chemistry, Gothenburg, Sweden

**Keywords:** TERRA lncRNA, R-loop, Non-telomeric DNA binding, Gene Regulation

## Abstract

Telomeres are transcribed into telomeric repeat–containing RNA (TERRA), a long non-coding RNA (lncRNA) with established roles in telomere maintenance and genome stability. While TERRA’s formation of R-loops at telomeric regions is well documented, its potential interactions with non-telomeric DNA remain less clear. Here, through bioinformatic reanalysis of publicly available datasets, we identify evidence that TERRA may also associate with non-telomeric genomic regions in trans via R-loop formation. These interactions are enriched at loci containing tandem telomeric repeat motifs, suggesting a sequence-dependent mode of binding. Integrative analysis further indicates that such distal TERRA–DNA associations could influence the expression of telomere maintenance–related genes, hinting at a possible gene-modulating role. While based on bioinformatics analysis, these findings generate testable hypotheses that warrant experimental validation and open new avenues for exploring how TERRA, and potentially other lncRNAs, contribute to transcriptional regulation and chromatin organization.

## Graphical Abstract

**Figure:**
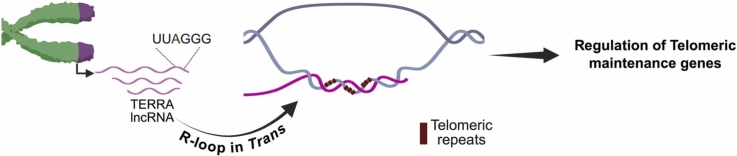

## 1 Introduction

TERRA (telomeric repeat-containing RNA), a lncRNA originating from sub-telomeric regions extending towards telomere ends, was first reported 25 years ago in *Trypanosoma brucei* [1]. In humans, TERRA contains UUAGGG telomeric repeats and was first reported in 2008. TERRA is a heterogeneous population of lncRNAs, varying widely in length (100 bases to >9 kb) [2], and may or may not possess polyA tails in the mature RNA transcripts [3]. Despite over two decades of study, many aspects of TERRA remain to be understood.

Telomere maintenance primarily depends on the activity of the telomerase enzyme encoded by the TERT gene. However, a telomerase-independent alternative mechanism known as Alternative Lengthening of Telomeres (ALT) also exists. Although the exact mechanisms driving cells towards ALT are not fully understood, inactivating mutations in the alpha thalassemia/mental retardation syndrome X-linked (ATRX) gene are thought to be a key factor. We and others have shown that TERRA is highly expressed in various cancers positive for ALT [4], [5], [6]. This aligns with the notion that ATRX antagonizes TERRA [7]. Studies in yeast where TERRA was upregulated revealed cells with reduced senescence and longer telomeres [8], [9]. TERRA forms R-loops (RNA-DNA hybrid structures) in cis at the telomere ends where it is transcribed, playing critical roles in telomere maintenance and elongation [4], [10], [11], [12], [13].

While telomere maintenance by TERRA suggests its cis-acting function, it has been proposed that TERRA also functions in trans. Using TERRA CHIRT sequencing (Chromatin Isolation by RNA Purification, ChIRP, Capture Hybridization Analysis of RNA Targets, CHART), TERRA binding was mapped across the entire mouse genome. Interestingly, TERRA binding to non-telomeric regions of the genome was also identified in a significant proportion [7], [14]. This suggests either a role for TERRA outside of telomere maintenance or that TERRA exerts telomere maintenance by binding non-telomeric regions of the genome.

Using a TERRA reporter system, Feretzaki et al. demonstrated that trans-acting TERRA is targeted to telomere ends by forming R-loop structures with the help of proteins such as RAD51 and BRCA2 [13]. In mouse embryonic stem cells, mapping TERRA binding sites showed that TERRA is also targeted to non-telomeric genomic regions across different chromosomes [7], [14], [15]. Importantly, how TERRA interacts with its non-telomeric genomic targets in trans remains largely unknown [7].

In this study, we bioinformatically reanalysed genome-wide R-loop [16] (DNA–RNA immunoprecipitation followed by sequencing, DRIP-seq), and TERRA binding [7] (TERRA CHIRT-seq) datasets from mouse embryonic stem cells to investigate whether trans (non-telomeric) TERRA localization is driven by R-loop formation. We found that TERRA is preferentially enriched at genomic loci harboring R-loops, suggesting that R-loop structures may guide TERRA targeting in trans. This mechanism could represent a broader strategy used by other trans-acting lncRNAs to modulate the expression of their target genes. If generalizable, such an R-loop–linked distribution may serve as a means by which trans-acting lncRNAs are positioned to influence gene regulation across the genome.

## 2 Materials and methods

### 2.1 Analysis scripts

All analyses were performed using publicly available tools and custom scripts, as detailed below. Exact commands, parameters, and workflow scripts are available in the accompanying GitHub repository: https://github.com/saifshehata/terra_rloop. Scripts are named and commented descriptively to clearly indicate the specific analysis step or figure they correspond to.

### 2.2 Raw data accession numbers and data visualization

TERRA CHIRT-Seq (anti-sense: SRR2062968, sense control: SRR2062969), R-loop DRIP-Seq (SRR2075686), and ATRX ChIP-Seq (SRR057567) sequencing files were obtained from the Short-Read Archive (SRA) database [17] using the Gene Expression Omnibus (GEO) accessions for TERRA (GSE79180), R-loop (GSE70189), and ATRX (GSE22162) using the Entrez Direct E-utilities [18].

All bar-plots, boxplots, karyo-plots and Venn diagrams were generated using ggplot2 v3.3.6 and KaryoplotR v1.18.0 packages in R [19], [20]. All metagene coverage profiles and heatmaps were generated using DeepTools v3.5.1 (computeMatrix, plotProfile, and plotHeatmap) [21]. All peaks were visualized using Gviz v1.36.2 (https://bioconductor.org/packages/release/bioc/vignettes/Gviz/inst/doc/Gviz.html, https://github.com/ivanek/Gviz) in R. Motif analysis was performed using MEME v5.4.1 [22], [23], and pie-charts were generated using both ggplot2 and Microsoft Excel.

### 2.3 Identifying TERRA and R-loop peaks

Sequencing reads in FastQ format were retrieved from the SRA database using Sra-Toolkit v2.11.0 (fastq-dump) developed by the SRA Toolkit Development Team at [24]. After quality control using FastQC v0.11.9 [25], sequences with a Per Base Sequence Quality of less than 28 were trimmed using Trimmomatic v0.39 [26]. The resulting reads were aligned to the mouse mm10 genome using BWA-MEM v0.7.17 [27], and peaks were identified using MACS2 (callpeak) v2.2.7.1 [28], [29] using default parameters, while specifying the mappable genome size (-g mm) and whether the input BAM files were single (-f BAM) or paired-end (-f BAMPE).

### 2.4 Identifying overlapping peaks

Narrow Peak files from MACS2 peak calling for both TERRA and R-loop were used as input to BEDTools v2.30.0 (intersectBed) [30] to obtain peaks that contained a minimum of one base pair overlap/intersection.

### 2.5 Annotating peaks and counting telomeric repeats per peak

The overlapping TERRA peaks (identified using intersectBed) were annotated with HOMER v4.11 (annotatePeaks.pl) to identify the gene with the nearest transcription start site (TSS) to each peak. We additionally used the –nmotifs and –mbed parameters [31], [32] to identify the number and location of telomeric repeats within each peak. Custom Bash scripts were then used to identify and extract peaks with a minimum of 4 tandem telomeric repeats, as well as to retrieve the longest stretch of tandem repeats per peak. The resulting annotation files were imported into R for downstream analysis, including counting and visualizing telomeric repeats per peak.

### 2.6 Peak classification based on telomeric repeat content

Peaks were classified into five types (I-V) based on the abundance and distribution of telomeric repeat motifs (TTAGGG or its reverse complement CCCTAA) within their genomic coordinates (Fig. 2D). Type (I) peaks lacked any telomeric repeats, type (II) contained a single repeat, and type (III) harboured multiple but dispersed repeats without any continuous stretch of four or more tandem units. Type (IV) peaks contained at least one stretch of four tandem repeats, whereas type (V) contained multiple such stretches.

### 2.7 Metagene coverage profiles and heatmaps

Read coverage was computed using computeMatrix, plotProfile and plotHeatmap from DeepTools https://deeptools.readthedocs.io/en/latest/). Alignment bigwig files were used as input score files and intersecting peak BED files as the input regions files. Regions files included R-loop peaks that intersected with TERRA peaks split by presence or absence of tandem telomeric repeats, as well as by genome annotation (intron or intergenic). To enable direct visual comparison, ATRX and R-loop heatmaps were sorted using the peak order derived from the TERRA heatmap. Peaks that lay within the ENCODE blacklisted regions (https://github.com/Boyle-Lab/Blacklist/raw/master/lists/Blacklist_v1/mm10-blacklist.bed.gz) or that were within subtelomeric regions (defined as being 500 kb from chromosome ends) [33], [34] were uniformly excluded from all the datasets of the resulting profiles and heatmaps. Removal was achieved by adding a file name with the blacklist and subtelomere locations to the computeMatrix command using the '--blackListFileName' parameter.

### 2.8 Permutation analysis of TERRA–R-loop overlap significance

To assess the statistical significance of the observed overlap between TERRA and R-loop peaks, we conducted a permutation (randomization) test. In this analysis, the genomic coordinates of TERRA peaks and R-loop peaks were each randomized independently across the mappable genome while preserving their original number, size, and chromosome distribution. For each of 1000 permutations, the number of overlapping peaks between the randomized TERRA and R-loop datasets was calculated using the same intersection criteria applied in the original analysis (an overlap of at least one nucleotide). The resulting distribution of overlap counts from the randomized datasets was then compared to the observed overlap from the real data. The empirical p-value was estimated as the proportion of permutations in which the randomized overlap count was equal to or greater than the observed overlap.

## 3 Results

### 3.1 A small fraction of TERRA and R-loop peaks overlap

**Fig. 1:**
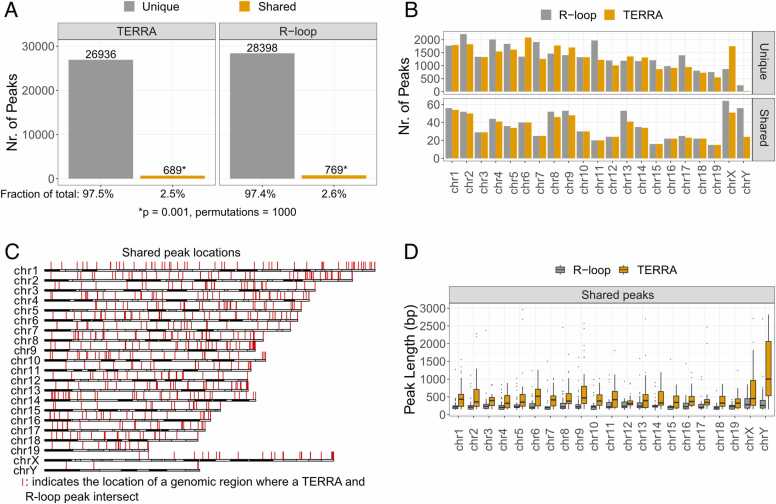
A small fraction of TERRA and R-loop peaks overlap. A) Total number of unique TERRA peaks after peak calling, as well as those shared (overlapping) with R-loops, and vice versa. A hypermutation test was performed to assess statistical significance of the shared peaks relative to the total. B) Number of TERRA and R-loop peaks per chromosome shown separately for shared and unique peaks. C) Genomic positions of shared peaks displayed across each chromosome. D) Distribution of shared TERRA and R-loop peak lengths per chromosome.

To determine whether TERRA RNA may bind to non-telomeric DNA sites in trans via R-loops, we obtained published datasets containing sequencing reads after TERRA [7] or R-loop [16], [35] immunoprecipitation from mouse embryonic stem cells. After processing and aligning the reads to the mouse (mm10) genome, we identified peaks to determine the genomic coordinates where TERRA and R-loops are most likely located. To identify the maximum number of potential loci, we chose not to filter the peaks by fold enrichment, resulting in approximately 28,000 TERRA peaks and 29,000 R-loop peaks (Fig. 1A). Notably, filtering for peaks with a fold enrichment of at least 10 over control resulted in approximately 4000 TERRA peaks, confirming results by Chu et al. [7].

If TERRA indeed binds its DNA targets via R-loops, their locations on the genome should coincide, leading to overlapping/intersecting peaks in genomic regions. To maximize the number of intersecting peaks, we filtered for TERRA and R-loop peaks that contained an intersection of at least one base pair. A small fraction (∼3 %) of TERRA peaks overlapped with R-loop peaks (‘shared’ peaks), whereas the majority did not (‘unique’ peaks) (Fig. 1A). Throughout the manuscript, we use ‘shared’ and ‘unique’ specifically to refer to TERRA peaks that do or do not intersect R-loop peaks, unless explicitly stated as ‘shared R-loop peaks’, in which case we refer to the R-loop peaks intersecting TERRA peaks.

To evaluate whether the observed overlap between TERRA and R-loop peaks was greater than expected by chance, we performed a permutation-based significance test (see *Methods*). The analysis demonstrated that the observed number of shared peaks was significantly higher than in any of the randomized datasets (Fig. 1A and S1A), confirming that the TERRA–R-loop intersections, though ∼3 % of total peaks, are highly unlikely to have occurred by random distribution across the mappable genome.

The significance of the overlap between TERRA and R-loop peaks prompted further investigation into their genomic distribution. Examination of the chromosomal distribution of shared peaks revealed that they were present in comparable numbers across all chromosomes, indicating no enrichment or bias toward any specific chromosome (Fig. 1B).

Given that TERRA is transcribed from telomeric regions, we next assessed whether shared peaks were preferentially enriched at telomeric or sub-telomeric loci. Analysis of shared peak distributions along individual chromosomes revealed modest enrichment near the distal ends of a subset of chromosomes (e.g., 9, 10, 11, and 13), but overall, shared peaks were broadly distributed across chromosomal arms (Fig. 1C).

R-loops are generally formed co-transcriptionally and, depending on gene length, can span extensive regions along actively transcribed loci [36]. To assess whether the TERRA-associated R-loops exhibited such characteristics, we compared R-loop and TERRA peak lengths across chromosomes. R-loop peaks were found to be relatively narrow, with an average length of ∼150–350 bp (Fig. 1D), suggesting that these structures likely represent distal or trans interactions of TERRA rather than co-transcriptional R-loop formation. Interestingly, shared TERRA peaks were, on average, broader than the corresponding R-loop peaks across all chromosomes (Fig. 1D). In many cases, R-loop signals were fully contained within TERRA-associated regions, while some TERRA domains encompassed multiple discrete R-loop peaks (data not shown).

### 3.2 Telomeric repeats are highly enriched in shared peaks

**Fig. 2:**
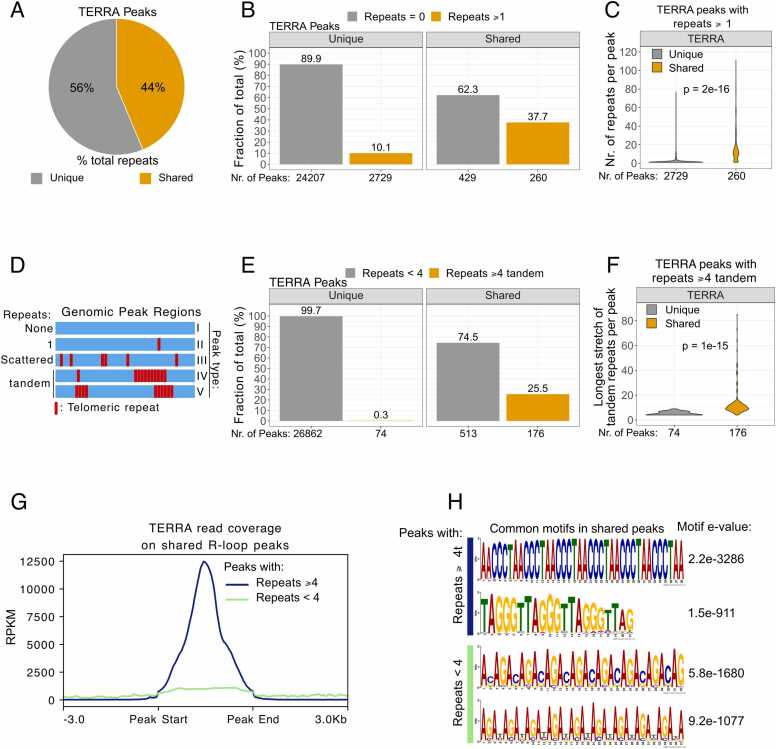
Telomeric repeats are highly enriched in shared peaks. A) Fraction of all telomeric repeats that are located in shared vs. unique peaks. B) Fraction of shared and unique peaks that either contain or lack at least one telomeric repeat. C) Distribution of telomeric repeat counts across shared and unique peaks harbouring at least one telomeric repeat, with the means of the two groups compared using a *t*-test. D) Categorization of peaks into five groups based on the number and distribution of telomeric repeats (see *Methods* section for more details). E) Fraction of shared and unique peaks that either contain or lack four tandem telomeric repeats. F) Distribution of the longest stretch of telomeric repeats per peak across shared and unique peaks harbouring at least four telomeric repeats, with the means of the two groups compared using a *t*-test. G) Coverage profiles of TERRA sequencing reads over genomic regions shared with R-loops that contain or lack four tandem telomeric repeats. H) Most common sequence motifs in shared peaks that contain or lack four tandem telomeric repeats.

A defining feature of TERRA is the presence of long stretches of short tandem telomeric repeat sequences (5’-UUAGGG-3’). However, telomeric repeats are also found at non-telomeric sites across the genome [7]. The presence of telomeric repeats in both TERRA and genomic regions should, in principle facilitate R-loop formation due to sequence complementarity. To investigate this, we counted the total number of single telomeric repeats in all TERRA peaks and calculated their distribution across shared and unique peaks. Surprisingly, 44 % of all telomeric repeats were enriched in the shared peaks that represented less than 3 % of all TERRA-associated regions. On the other hand, all remaining unique peaks harboured 56 % of telomeric repeats (Fig. 2A). This hinted that TERRA RNA targeting in trans may be achieved via telomeric repeat-dependent R-loop formation.

Next, we filtered TERRA peaks that contained at least one telomeric repeat. We found that while ∼38 % of shared peaks (260 out of 689 peaks) contained at least one repeat, ∼10 % of unique peaks (2729 out of 26936 peaks) contained at least one repeat (Fig. 2B). We then investigated the frequency of these telomeric repeats per peak. Interestingly, the majority of the shared peaks (82 %, 212 of 260 peaks) had more than one telomeric repeat, with a median of 11 repeats per peak. On the other hand, close to 81 % (2215 of 2729) of unique peaks had only one telomeric repeat (Fig. 2C), with a median of one repeat per peak, suggesting that TERRA peaks that intersect with R-loops have a distinct enrichment of consecutive/tandem telomeric repeats.

To explore this further, we assessed the repeats within all TERRA peaks (shared and unique) and performed peak classification based on repeat content (Fig. 2D). *In vitro* experiments suggest that a requirement of three tandem telomeric repeats is sufficient for R-loop formation [37]. Based on this information we considered that a minimum of four tandem telomeric repeats (i.e. TTAGGG x 4 or CCCTAA x 4) would be needed for the formation of stable R-loops in cells (Fig. 2D, peak types IV & V). Therefore, we filtered for TERRA peaks with a stretch of at least four tandem telomeric repeats, and found that while a quarter (∼25 %) of shared peaks passed this filter, the vast majority (99.7 %) of unique peaks lacked tandem repeats (Fig. 2E), further confirming the high enrichment of long stretches of telomeric repeats only in TERRA peaks that intersected with R-loops. To further validate this, we counted the longest stretch of tandem repeats in each TERRA peak, while excluding peaks with less than four tandem repeats, and found that only shared peaks contained long stretches of tandem repeats (Fig. 2F). This confirms that although many unique TERRA peaks contained a high number of telomeric repeats (Fig. 2C), the vast majority of these repeats are scattered (Fig. 2D, peak type III) and are likely unable to form R-loops in vivo. This data suggested that the presence of tandem telomeric repeats in genomic DNA can serve as a predictor of TERRA-dependent R-loop formation.

We noted that ∼75 % of shared peaks (513 out of 689) lacked tandem telomeric repeats (Fig. 2E), which prompted further investigation. We asked if the presence of telomeric repeats in the shared peaks correlated with TERRA enrichment and found that TERRA enrichment was 10-fold higher in peaks with four or more tandem repeats (Fig. 2G). This indicated that TERRA preferentially associates with telomeric repeat-enriched genomic regions. Furthermore, to assess the effectiveness of our four-tandem-repeat filter, we performed a motif analysis on the peak regions. This would enable us to better understand why many TERRA peaks lacking tandem repeats still intersected R-loop peaks, though with markedly reduced coverage. Our motif analysis confirmed that tandem telomeric repeats (TTAGGG or CCCTAA) were only found in those peaks that passed our filter, while peaks that lacked tandem telomeric repeats were enriched in AG-rich motifs (Fig. 2H). The presence of AG-rich regions may support R-loop formation independent of TERRA, as was the case in several such genomic regions (Fig. S2A), especially since R-loop presence was not exclusive to telomeric repeat-containing peak regions (Fig. S2B). Collectively, these results implicate the presence of stretches of tandem telomeric repeats as predictors of TERRA-dependent R-loop formation.

### 3.3 ATRX coverage is analogous with TERRA

**Fig. 3:**
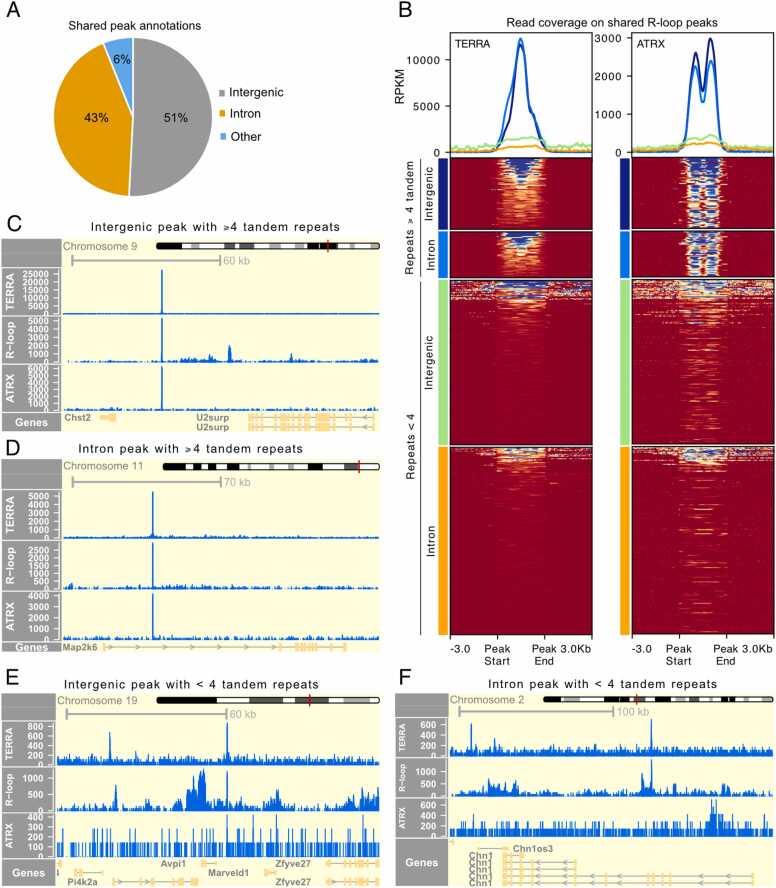
ATRX overlaps with shared TERRA–R-loop peaks, but not within peaks lacking tandem telomeric repeats. A) Genomic annotation of shared peaks, shown as the percentage of peaks located in intronic, intergenic, or other genic regions (promoter–TSS, TTS, 3′UTR, exons). B) Metagene profiles and heatmaps depicting TERRA and ATRX coverage across intronic and intergenic regions shared with R-loops, with peaks grouped by those that contain or lack four tandem telomeric repeats. C–D) Genome browser views of TERRA, R-loop, and ATRX coverage at representative intergenic (C) and intronic (D) peaks containing at least four tandem telomeric repeats. E–F) Genome browser views of TERRA, R-loop, and ATRX coverage at representative intergenic (E) and intronic (F) peaks lacking four tandem telomeric repeats.

The regulation of gene transcription is a complex process involving many factors, and it is conventionally thought to be regulated primarily by transcription factor binding to promoter sequences [38]. Another major regulator of gene transcription is enhancer sequences, which can be up to tens of megabases away from the gene promoter yet still regulate transcription [39]. We observed that the majority (over 90 %) of shared peaks were located either within introns (∼43 %) or in intergenic regions (∼51 %) (Fig. 3A). When assessing the relative enrichment of TERRA sequencing reads on shared R-loop peak regions, we found that peaks with tandem repeats had significantly higher coverage than those lacking them, regardless of whether the peaks were within introns or intergenic regions (Fig. 3B).

Furthermore, it was recently shown that ATRX, a chromatin remodeler, interacted with TERRA at many genomic loci [7], where the authors demonstrated that ATRX competes with TERRA for DNA binding to regulate telomere maintenance and protect telomeres [7]. We utilized a publicly available ATRX ChIP-seq dataset [40] and found a high relative enrichment of ATRX signal at shared peaks containing tandem telomeric repeats (Fig. 3B). To further investigate whether this enrichment of TERRA and ATRX was biased toward specific genomic annotations, we divided the peak regions in the heatmap according to their genomic annotation (intron or intergenic). We observed that the presence of telomeric repeats, rather than genomic annotation, was the main factor associated with ATRX enrichment at these shared peak sites (Fig. 3B). The presence of high ATRX signal over repeat-enriched shared peaks suggested that ATRX/TERRA-driven R-loop formation at these loci might have a regulatory role in gene expression.

To further validate this, we visualized individual shared peaks that lay within telomeric repeat-enriched genomic regions and observed stronger signals for TERRA, R-loop, and ATRX peaks in both intronic and intergenic regions (Fig. 3C and D) compared to those that were not repeat-enriched (Fig. 3E and F). We also observed broad R-loop peaks spanning several exons within genes, for instance in the Death-associated protein kinase 1 (Dapk1) and BRCA1-associated RING domain protein 1 (Bard1) genes (Fig. S3A and B). Interestingly, in the peaks within Dapk1 and Bard1, the R-loops associated with TERRA, though surrounded by broader R-loop regions, were more highly enriched and spanned a much narrower region that overlapped with TERRA peaks. This distinction from the surrounding R-loops further supported the notion that these stable R-loops are a result of the trans binding of TERRA.

### 3.4 A small subset of genes associated with shared peaks are differentially expressed upon TERRA knock-down

**Fig. 4:**
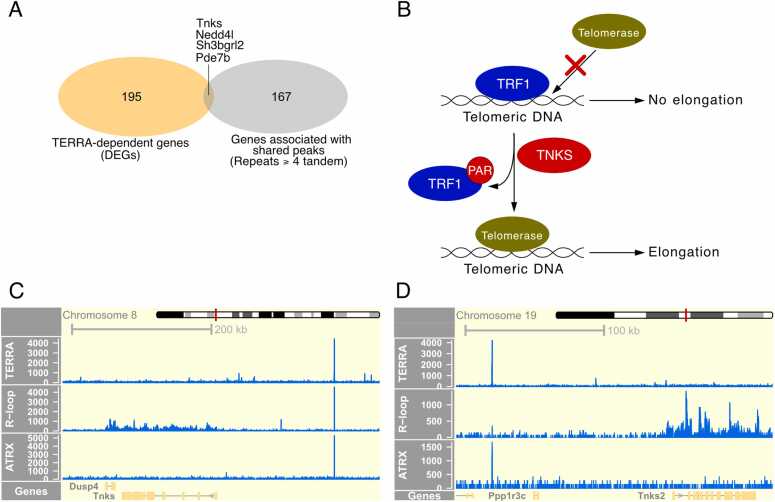
A portion of genes downregulated upon TERRA knockdown are associated with nearby shared TERRA–R-loop peaks enriched in tandem telomeric repeats. A) Overlap between genes downregulated upon TERRA knockdown and genes associated with repeat-enriched shared peaks. B) Overview of the opposing effects of TRF1 (TERF1) and TNKS1 on telomere elongation. C–D) Genome browser views of TERRA, R-loop, and ATRX coverage at selected genes from the overlap in panel A.

It was previously reported that a number of genes were differentially expressed upon TERRA knockdown [7]. Although the downstream effects of TERRA binding to DNA/protein involve adapter functions [35] and may not be limited to direct modulation of gene transcription, we wanted to explore whether any genes associated with our shared TERRA peaks were previously reported to be differentially expressed upon TERRA knockdown [14]. When we extracted genes associated with or in close proximity to tandem repeat-enriched shared peaks (Supplementary Table S1), we observed that a small subset of genes overlapped with those previously reported as differentially expressed in a TERRA-dependent manner (Fig. 4A).

Interestingly, one of these genes was Tankyrase 1 (TNKS1), a Poly-ADP-Ribosylation polymerase (PARP) known to regulate telomere elongation by PARsylation of Telomeric Repeat Binding Factor 1 (TRF1) [41], [42], [43], [44]. PARsylation of TRF1 results in its release from telomeric DNA and subsequent degradation via ubiquitylation, allowing telomerase to elongate telomeres (Fig. 4B) [45], [46]. It would be interesting to explore potential regulation of TNKS1 by TERRA, and whether this may in turn affect telomere length under certain physiological conditions.

Moreover, we detected shared peaks within 130–170 kb of the promoter regions of both TNKS1 and TNKS2 (Fig. 4C and D), both of which contribute to telomere maintenance. This may suggest a possible avenue for future investigation into whether TERRA may influence their expression.

Taken together, our results suggest a possible mechanism by which TERRA RNA may act in trans by forming R-loop structures at genomic regions enriched in tandem telomeric repeats. This highlights avenues for future research into potential transcriptional regulation of genes that harbour these R-loops in their vicinity. However, these conclusions are based on computational analyses, and dedicated experimental validation will be required to test this model. Our findings further raise the possibility that such R-loop–mediated regulation could represent a more general mechanism employed by other trans-acting lncRNAs to modulate the expression of their target genes.

## 4 Discussion

TERRA and other lncRNAs have previously been shown to bind non-telomeric genomic regions [7], [47]. In this study, by reanalysing TERRA and R-loop datasets from mouse embryonic stem cells, we present evidence that TERRA (telomeric repeat–containing RNA) is enriched at non-telomeric genomic sites containing tandem telomeric repeat motifs, where its presence strongly correlates with R-loop formation. Our data suggest that TERRA may be targeted to non-telomeric regions via R-loop–mediated interactions with DNA. This is supported by our observation of a 10-fold higher TERRA enrichment in tandem repeat-containing peak regions (Figs. 2G and 3B). Interestingly trans targeting of the lncRNA APOLO in plants is also guided by R-loop formation [48]. In addition, the lncRNA MEG3 has been shown to target trans genomic regions via direct RNA–DNA triplex binding, a process that depends on GA-rich motifs [47]. These observations suggest that targeting of a lncRNA in trans via direct interaction with DNA sequences could be a common mechanism [49]. Moreover, Feretzaki et al. reported that plasmid-derived TERRA can be targeted in trans to telomeres from which they did not originate, a process mediated by RAD51/BRCA2-dependent strand invasion and R-loop formation [13]. Based on our reanalysis of published genomic TERRA and R-loop datasets, we extend this concept of trans (telomere-to-telomere) TERRA-dependent R-loop formation to non-telomeric regions. While our data suggest that TERRA may form R-loops at repeat-enriched non-telomeric loci, this evidence is correlative, and experimental approaches are needed to identify other molecular players which guide such R-loop formation. It will be interesting in future work to determine which proteins mediate strand invasion at non-telomeric loci, including factors like RAD51, and to assess their involvement in TERRA-mediated R-loop formation. Furthermore, it is conceivable that 3D telomere looping [50], [51], [52] could potentially reduce the spatial distance TERRA must traverse to form trans R-loops at the non-telomeric genomic regions.

It is also important to note that only a fraction of TERRA-binding sites overlap with R-loops, suggesting that additional mechanisms, such as RNA-protein interactions [53], may guide TERRA to non-telomeric genomic regions lacking telomeric repeat enrichment. Of further interest was our finding that a significant portion of shared peaks did not contain telomeric repeats (Fig. 2B). We therefore cannot exclude the likelihood of TERRA RNAs forming R-loops at genomic regions that lack telomeric repeats. One explanation may be the presence of AG-rich repeats within these regions (Fig. 2H), which may partially mimic the G-rich telomeric repeat, providing a weak template for complementary binding and R-loop formation. Another explanation might be that these genomic regions share high sequence similarity with the sub-telomeric portions of many TERRA RNAs, enabling them to more easily form stable R-loops [13].

Additionally, we show that the chromatin remodelling factor and RNA binding protein ATRX is enriched at shared peaks. This aligns with previous work showing that TERRA and ATRX share common target genes and that TERRA binding may influence ATRX-DNA binding [7]. More recently, it has been demonstrated that TERRA depletion resulted in a reduction of DNA G-quadruplex (G4) structures at transcription start sites, leading to enhanced ATRX binding [14]. In our study, we provide evidence that strong TERRA-binding sites enriched with telomeric repeats are also enriched in R-loops. These R-loops may colocalize with G4 structures, thereby promoting the formation of G-loop structures as reported recently [54]. G-loops are generated when an R-loop forms on the strand opposite to a G4 structure through strand invasion mediated by BRCA2- and RAD51-like proteins. Notably, G4 and R-loop structures are often co-localized in the genome [54], [55], suggesting functional interdependence between the two. Consistent with this idea, the decrease in G4 levels observed upon TERRA depletion [14] could reflect a reduction in trans R-loops in the absence of TERRA, ultimately destabilizing G4 structures. Further studies will be required to determine how TERRA-dependent G4/R-loop/ATRX interactions influence gene expression.

We observed that only a small fraction of genes associated with shared peaks exhibited TERRA-dependent changes in gene expression. Among these is TNKS1, a gene involved in telomere maintenance [41], [43], [45]. This observation raises the possibility that R-loop–mediated trans TERRA binding could play a role in regulating genes important for telomere biology. However, the molecular mechanisms underlying such TERRA-dependent regulation remain to be elucidated. It is important to emphasize that the overlap between TERRA-regulated genes and genes associated with shared peaks is limited, and several factors may contribute to this. Previous studies have shown that TERRA-dependent gene regulation can involve epigenetic mechanisms [15]. Since the TERRA depletion used in these datasets was transient (12 h) [14], such a short timeframe may be insufficient to reverse epigenetically driven regulation, resulting in restricted overlap. Another factor may be the challenge of achieving complete TERRA knockdown, as it remains unclear whether residual levels of TERRA may still exert significant regulatory effects. Together, these limitations may contribute to the modest overlap observed between TERRA-regulated genes and genes associated with shared TERRA peaks [7].

In conclusion, our bioinformatic reanalysis of published datasets suggests that TERRA may associate with non-telomeric distal regions of the genome through R-loop formation. Although the molecular mechanisms driving such trans targeting remain to be thoroughly validated with more robust experimental data, these findings raise the possibility that long-range genomic targeting by lncRNAs may not be limited to TERRA. Instead, it could represent a more general mechanism employed by other lncRNAs to regulate gene expression.

## Author statement

Saifeldin N. Shehata, Akram Mendez, Tanmoy Mondal, and Roshan Vaid all contributed substantially to this work. The study was conceived and designed by Tanmoy Mondal and Roshan Vaid, who also led the biological interpretation of the results and contributed to securing funding for the project. Saifeldin N. Shehata performed the bioinformatic analyses, with guidance from Akram Mendez. The manuscript was drafted by Saifeldin N. Shehata and Roshan Vaid, and all authors contributed to the critical revision of the text, approved the final version of the manuscript, and agree to be accountable for all aspects of the work. Roshan Vaid serves as the corresponding author.

## CRediT authorship contribution statement

**Roshan Vaid:** Writing – review & editing, Writing – original draft, Supervision, Resources, Project administration, Investigation, Funding acquisition, Data curation, Conceptualization. **Tanmoy Mondal:** Writing – review & editing, Writing – original draft, Supervision, Resources, Project administration, Investigation, Funding acquisition, Data curation, Conceptualization. **Akram Mendez:** Supervision, Investigation, Formal analysis. **Shehata Saifeldin N:** Writing – review & editing, Writing – original draft, Visualization, Validation, Investigation, Formal analysis, Data curation.

## Declaration of Generative AI and AI-assisted technologies in the writing process

During the preparation of this work, the authors used ChatGPT (OpenAI) in order to refine scientific writing, improve clarity, and ensure consistency in the language of the manuscript. After using this tool/service, the authors reviewed and edited the content as needed and take full responsibility for the content of the publication.

## Funding

Postdoctoral grant from Svenska Sällskapet för Medicinsk Forskning (10.13039/501100003748SSMF) PG-22–0386 and Cancerfonden 230753PT to RV; Tore Nilsons Stiftelse, Assar Gabrielsson Fond, Sahgrenska University Hospital fond (Sahlgrenska Universitetssjukhusets stiftelser) to RV. Swedish Research Council [Vetenskapsrådet, 2018–02224]; Cancerfonden [22–2341 and 25 4538]; Barncancerfonden [PR 2019–077 and PR2025–0071]; Svenska Läkaresällskapet; Åke Wibergs Stiftelse; Kungl Vetenskaps- och Vitterhets-Samhället (KVVS) to TM and research positions grant from Barncancerfonden [TJ 2019–0077 and HFT2025–0013] to TM.

## Conflict of interest

The authors declare no competing interests.

## Data Availability

All scripts and code used in this study have been deposited in the following GitHub repository: https://github.com/saifshehata/terra_rloop

## Appendix A Supplementary material

Supplementary material

Permutation test using randomized peak regions to assess the significance of overlap between TERRA and R-loop peaks (see *Methods* section for more details)

A) Genome browser view of TERRA, R-loop, and ATRX coverage at an intronic peak enriched in AG repeats but lacking four tandem telomeric repeats. B) Metagene profile and heatmap of R-loop coverage at shared peaks located in intronic or intergenic regions, with peaks grouped by those that contain or lack four tandem telomeric repeats

A–B) Genome browser views of TERRA, R-loop, and ATRX coverage in intronic peaks with tandem telomeric repeats, flanked by broad R-loop peaks
