# Supplementary figures and images for: Telomeric repeat-containing lncRNA TERRA targets non-telomeric DNA in trans via R-loops
Source: Comput Struct Biotechnol J. 2025 Nov 29;27:5450–8. doi: 10.1016/j.csbj.2025.11.063 (PMC12720007; doi:10.1016/j.csbj.2025.11.063)

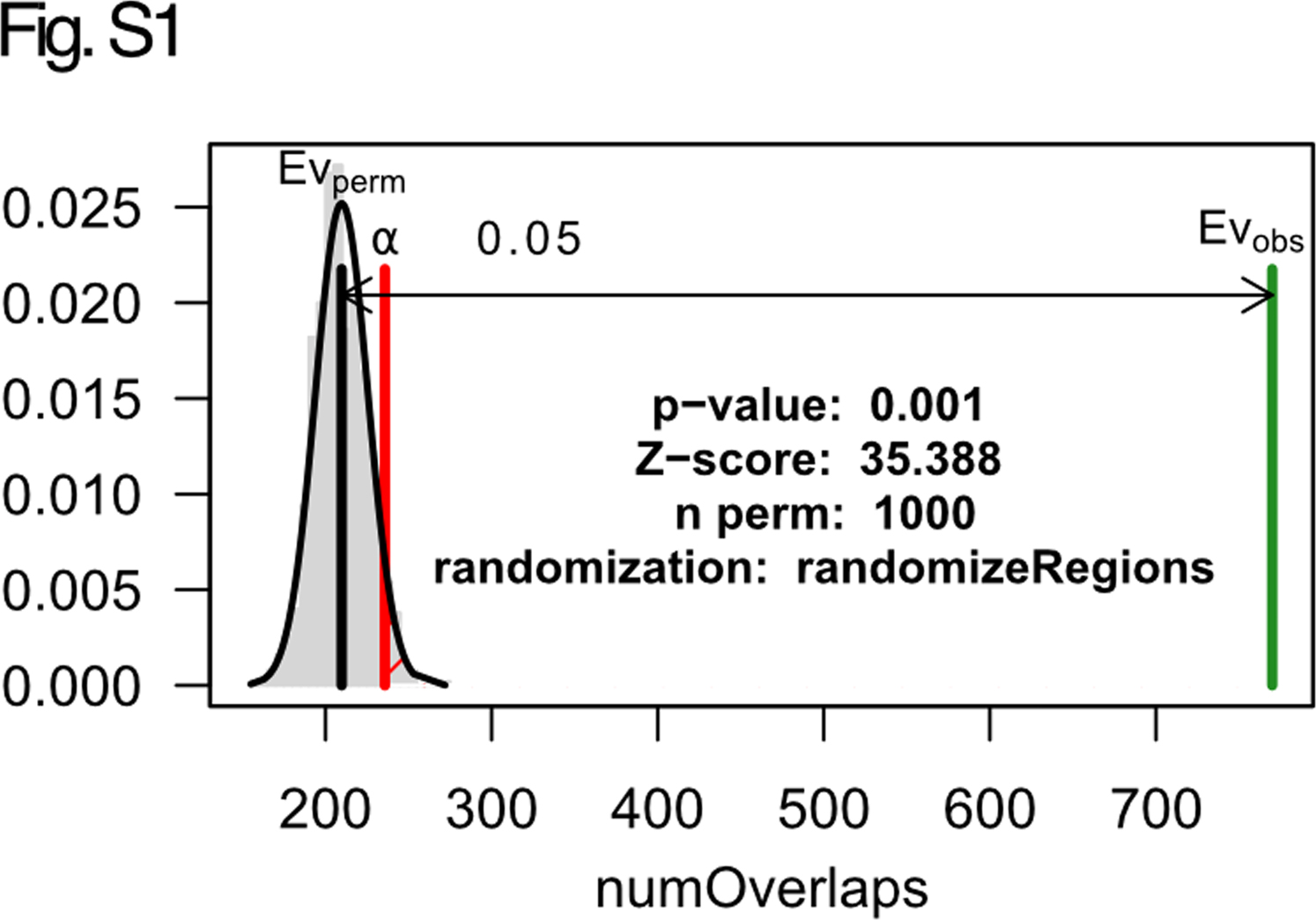

Supplement: Supplementary file 2 — Permutation test using randomized peak regions to assess the significance of overlap between TERRA and R-loop peaks (see Methods section for more details) [file mmc2.jpg]

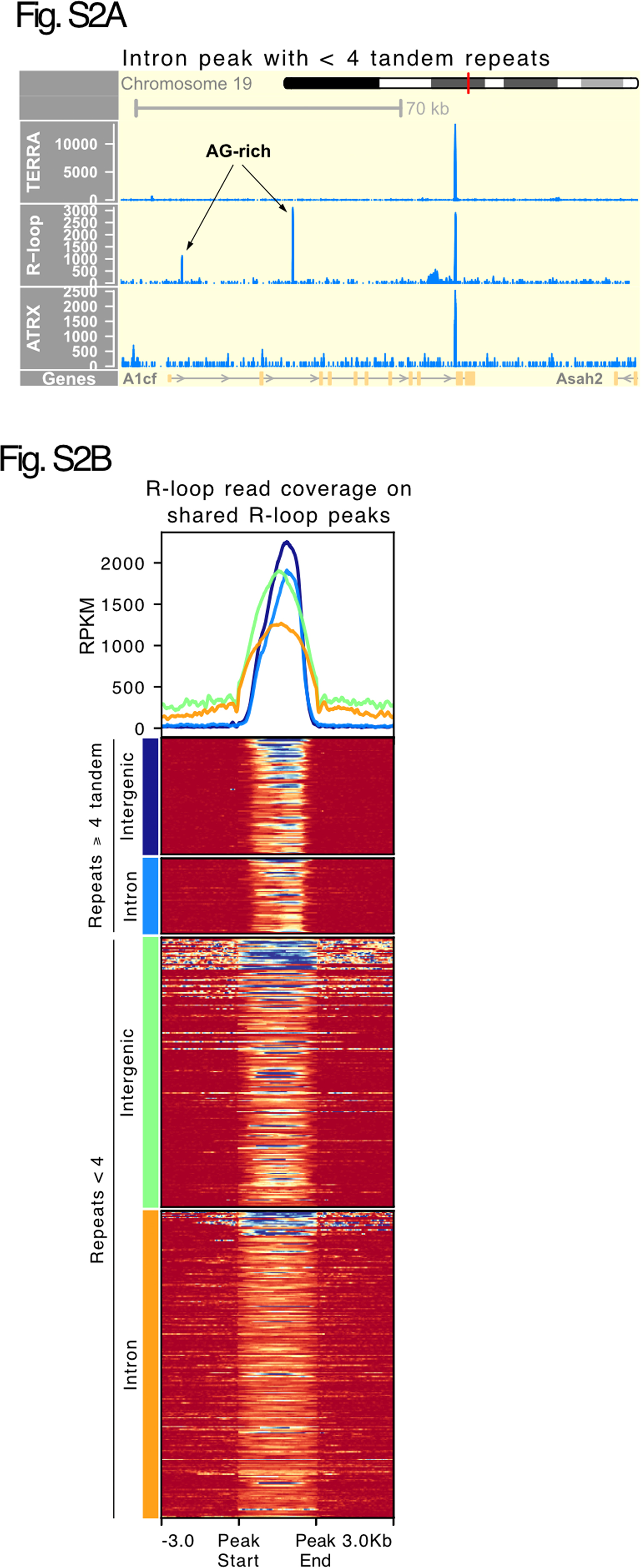

Supplement: Supplementary file 3 — A) Genome browser view of TERRA, R-loop, and ATRX coverage at an intronic peak enriched in AG repeats but lacking four tandem telomeric repeats. B) Metagene profile and heatmap of R-loop coverage at shared peaks located in intronic or intergenic regions, with peaks grouped by those that contain or lack four tandem telomeric repeats [file mmc3.jpg]

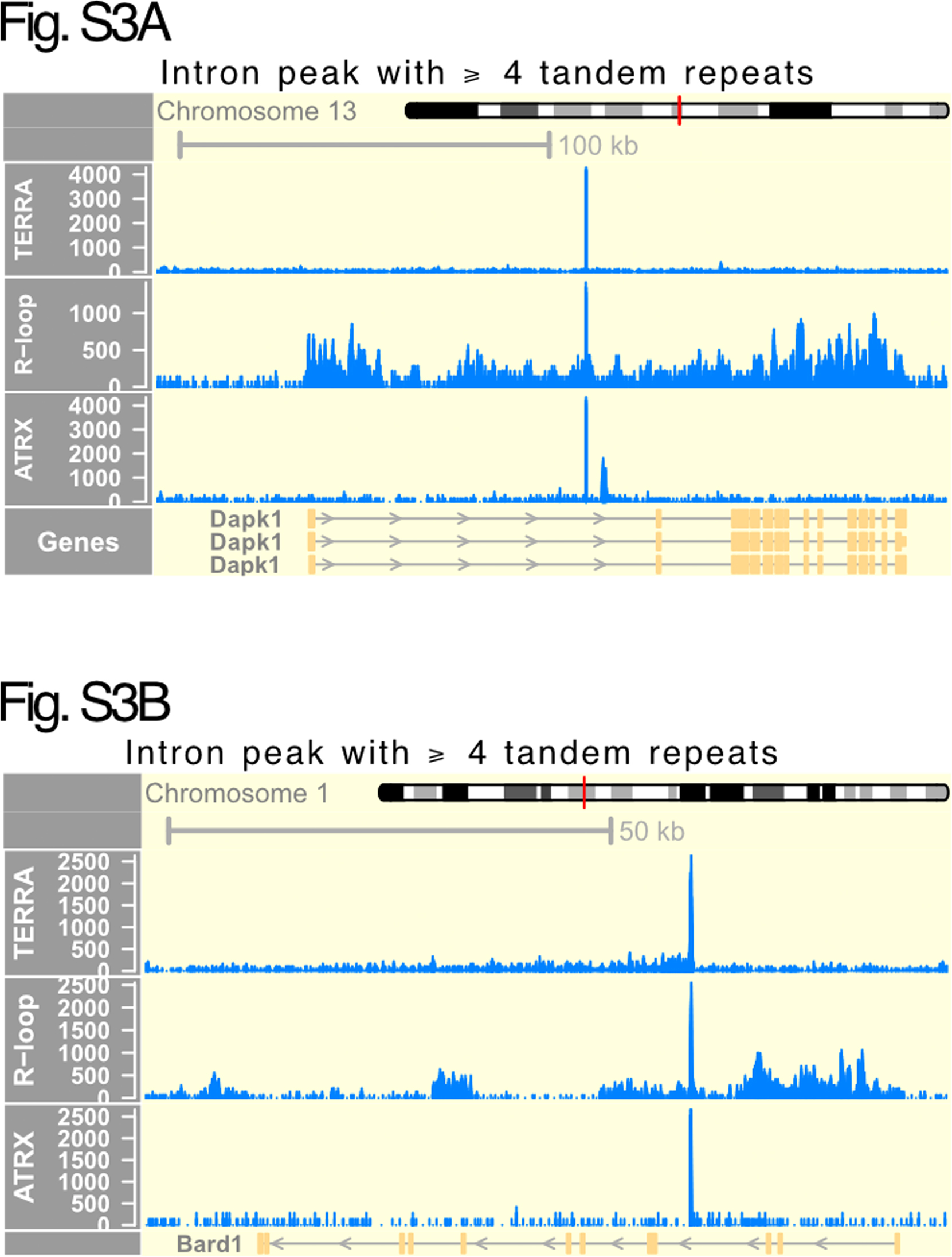

Supplement: Supplementary file 4 — A–B) Genome browser views of TERRA, R-loop, and ATRX coverage in intronic peaks with tandem telomeric repeats, flanked by broad R-loop peaks [file mmc4.jpg]
